# Interrogating the relevance of Ubuntu philosophy for disabilities in sub-Saharan Africa

**DOI:** 10.4102/ajod.v14i0.1619

**Published:** 2025-01-28

**Authors:** Mapheyeledi Motimele

**Affiliations:** 1 Department of Occupational Therapy, Faculty of Health Sciences, University of Cape Town, Cape Town, South Africa

Oliver Mutanga (ed.) and the contributors to this book are applauded for their critical insights and authenticity expressed through deliberately and intentionally centring their work on the affirmation of a shared humanity (Ubuntu) and the implications of centring this philosophy, for persons with disabilities, whose humanity remains in question and/or denied in society, despite legislation, policy and initiatives to address the status quo.

## From philosophy to practice

The data, perspectives and critical arguments about the potential for Ubuntu philosophy to promote critical disability scholarship and social inclusion in sub-Saharan Africa highlight Education, Governance and Social Development as sectors that are critical for social change at both the macro and micro levels. The authors’ contributions unpack how Ubuntu philosophy has the potential to guide initiatives or practical implementations of this philosophy, in our daily lives as educators, researchers, students, activists, policymakers and community members. Furthermore, the reader is challenged to critically reflect on the continued exclusion and underrepresentation of disability within everyday spaces where ‘humanity is enacted’ (Kronenberg 2018), despite efforts towards the contrary. What is the continued cost(s) of this and what are the structural, relational and agentic changes required to shift Ubuntu philosophy towards Ubuntu *practice*? Gore (2024), Mbazzi (2024), and Marovah and Mutanga’s (2024) chapters are helpful for their critical descriptions and considerations of what this may look like within Higher Education in South Africa, disability inclusive interventions in Uganda, and broader research within the Global South.

## Disability as an expression of coloniality

Regardless of one’s discipline, we should all be deeply concerned with the question of humanity. More specifically, ‘What does it mean to be human?’ and ‘What are the structures, systems and social arrangements that dehumanise or deny the humanity of some, while ‘authorising’ and thereby privileging the humanity of others?’ (Motimele 2024). From a decolonial perspective, the construct of ‘disability’ is deeply embedded within and impacted by coloniality, which continues to authorise and/or deny being through systems, structures and orientations that promote notions of inferiority and superiority, and therefore being/non-being (Fanon 1967). We have to grapple with these foundational questions if we are to understand the ways in which we (as both individuals and collectives) impact each other’s experiences of being/non-being, and how these experiences are expressions and manifestations of geopolitical relations of power (Grosfoguel 2006), especially as this relates to disability. Chinangaidze et al.’s (2024) chapter is insightful for their interrogation of the social, political, economic, environmental and technological relevance of Ubuntu philosophy towards disability inclusion across various sectors. Their analysis reinforces the wide-spread need for ‘ubuntu-centred’ notions of disability and inclusion that support a collective duty of care.

## What of an ‘ubuntu-informed’ disability model?

A common theme discussed by various authors in this book is the urgent need for a model of disability located within Ubuntu as a central philosophy, highlighting the need for centring indigenous knowledge systems within education policy and curricula (Ned 2019), especially as this relates to disability studies. Such a model holds potential to foreground the notion of humanity as an interconnected and intersectional experience, despite configurations of context that focus on separateness and reinforce social stratification. I encourage the authors to collectively consider how an Ubuntu-informed model of disability may look, thereby offering a critical tool for further engagement, interrogation and refinement.

Shandu-Phetla, Ngubane and Adigun’s (2024) chapter is especially thought-provoking for its consideration of technology and assistive technology, which, although widely considered as a social advancement and disability inclusion tool, risks further perpetuating disability exclusion, should the impact of disabling contexts, systems, structures, and relations, remain overshadowed by issues of race and gender in terms of equitable access to higher education institutions.

In conclusion, this book is highly recommended for disability scholars, educators, community leaders, health and social development practitioners and/or anyone concerned with social justice. This book encourages one to interrogate the ‘why’ and ‘how’ of their scholarship and/or practice and consider how they might anchor these in Ubuntu philosophy, while simultaneously navigating the risk of perpetuating disability exclusion through dominant disability perspectives, frameworks, methods, methodologies and interventions. What I most appreciated about this book was the opportunity it provided to ‘gather’ with advocates for Ubuntu-informed practice and scholarship in sub-Saharan Africa, through their research and critical insights. As a researcher, educator, occupational therapist and spiritual counsellor situated in the Global South, this book offered opportunity to reflect on my core beliefs and values and reimagine how these may need to shift to prioritise contextual relevance, disability representation and intersectionality as considered within an Ubuntu perspective, especially as this pertains to disability exclusion, marginalisation, erasure and dehumanisation.
